# Association between glycemic status and all-cause mortality among individuals with dementia: a nationwide cohort study

**DOI:** 10.1186/s13195-024-01557-x

**Published:** 2024-08-22

**Authors:** Youn Huh, Kye-Yeung Park, Kyungdo Han, Jin-Hyung Jung, Yoon Jeong Cho, Hye Soon Park, Ga Eun Nam, Soo Lim

**Affiliations:** 1 Department of Family Medicine, Uijeongbu Eulji Medical Center, Eulji University, Gyeonggi- do, South Korea; 2 Department of Family Medicine, Hanyang University College of Medicine, Seoul, South Korea; 3 Department of Statistics and Actuarial Science, Soongsil University, Seoul, South Korea; 4 Samsung Biomedical Research Institute, Sungkyunkwan University School of Medicine, Suwon, South Korea; 5 Department of Family Medicine, Daegu Catholic University School of Medicine, Daegu, South Korea; 6 Department of Family Medicine, Asan Medical Center, University of Ulsan College of Medicine, Seoul, South Korea; 7 Department of Family Medicine, Korea University Guro Hospital, Korea University College of Medicine, 148, Gurodong-ro, Guro-gu, Seoul, 08308 South Korea; 8 Department of Internal Medicine, Seoul National University Bundang Hospital, Seoul National University College of Medicine, 82, Gumi-ro 173 Beon-gil, Bundang-gu, Seongnam, 13620 South Korea

**Keywords:** Dementia, Diabetes mellitus, Prediabetes, Duration of diabetes, All-cause mortality

## Abstract

**Background:**

To examine the association between glycemic status and all-cause mortality risk among individuals with dementia.

**Methods:**

We enrolled 146,832 individuals aged 40 and older with dementia as identified through the Korean National Health Insurance Service health screening test between 2008 and 2016. Mortality status was evaluated at the end of 2019. Participants were classified into normoglycemia, prediabetes, or diabetes mellitus (DM) categories. The duration of diabetes was noted in those with DM. This study focused on the association between glycemic status and all-cause mortality.

**Results:**

The cohort, which was predominantly elderly (average age 75.1 years; 35.5% male), had a 35.2% mortality rate over an average 3.7-year follow-up. DM was linked with increased all-cause mortality risk (hazard ratio [HR] 1.34; 95% confidence interval [CI]: 1.32–1.37) compared to non-DM counterparts. The highest mortality risk was observed in long-term DM patients (≥ 5 years) (HR 1.43; 95% CI: 1.40–1.47), followed by newly diagnosed DM (HR 1.35; 95% CI: 1.30–1.40), shorter-term DM (< 5 years) (HR 1.17; 95% CI: 1.13–1.21), and prediabetes (HR 1.03; 95% CI: 1.01–1.05). These patterns persisted across Alzheimer’s disease and vascular dementia, with more pronounced effects observed in younger patients.

**Conclusions:**

Glucose dysregulation in dementia significantly increased mortality risk, particularly in newly diagnosed or long-standing DM. These findings suggest the potential benefits of maintaining normal glycemic levels in improving the survival of patients with dementia.

**Supplementary Information:**

The online version contains supplementary material available at 10.1186/s13195-024-01557-x.

## Background

Dementia, the most prevalent neurodegenerative condition globally, leads to progressive functional decline and affects approximately 55 million individuals worldwide in 2022. With an aging population, this number is expected to rise by 10 million new cases annually [1]. South Korea, a country experiencing rapid demographic aging, is witnessing an accelerated increase in dementia prevalence [2, 3]. Dementia currently ranks as the seventh leading cause of death worldwide and exacerbates the mortality risk 1.1–1.4 times more than cardiovascular events such as myocardial infarction and stroke [1, 4].

Given the absence of definitive treatments for dementia, the focus is on strategies to decelerate its progression [5]. Risk factors such as age, male sex, socioeconomic status, smoking, diabetes mellitus (DM), coronary heart disease, and psychiatric conditions have been identified to increase mortality in patients with dementia [6, 7]. In particular, DM is gaining attention because of its established role in elevating the risk of dementia onset [8, 9]. Due to aging and obesity, DM contributes to complications in vital organs, notably affecting the brain through cerebrovascular events and neuronal damage [10, 11]. Therefore, managing DM is essential for mitigating cerebrovascular complications and slowing dementia progression [5].

However, research on the effect of glucose regulation on the prognosis of dementia remains inconclusive. For instance, a U.S. study reported increased mortality in patients with Alzheimer’s disease having DM [12], while a Korean study noted a higher mortality risk in patients with Alzheimer’s disease having type 2 diabetes [13]. Conversely, a European study found no significant association between DM history and five-year mortality in patients with dementia [14]. These prior studies often involved small cohorts and relied on self-reported DM history instead of laboratory confirmation.

Our study aimed to clarify the relationship between glycemic regulation status and all-cause mortality in a large cohort of Koreans with dementia, providing empirical data on this significant medical concern.

## Methods

### Data source and study population

This study used nationwide cohort data obtained from the South Korean National Health Insurance Service (NHIS) (https://nhiss.nhis.or.kr/). The NHIS is a single universal insurer that covers nearly all South Koreans (approximately 50 million). The NHIS provides at least a biennial national health checkup for all South Koreans aged ≥ 40 years and all employees, regardless of age. Therefore, it includes comprehensive medical information about sociodemographic characteristics, health checkups (lifestyle and health examinations), and medical diagnosis and treatment based on the International Classification of Diseases, 10th revision (ICD-10) codes [15].

From this database, we initially identified 714,095 individuals who were newly diagnosed with dementia between January 1, 2008, and December 31, 2016. Dementia was defined based on ≥ 2 times/year of anti-dementia medication prescription (donepezil, rivastigmine, galantamine or memantine) under relevant ICD-10 codes (F00–F03, G30, or G31 for all-cause dementia). We further divided them into Alzheimer’s disease using F00 or G30 and vascular dementia using F01. In South Korea, anti-dementia medications can be prescribed when the test results of the Mini-Mental State Examination, Clinical Dementia Rating, or Global Deterioration Scale fulfill the dementia criteria [16, 17].

Among individuals newly diagnosed with dementia, we selected 165,430 individuals aged ≥ 40 years who underwent the NHIS health examination within 4 years after the diagnosis of dementia. We then excluded individuals with missing variables (n *=* 8,487) and those who died within 1 year of diagnosis (*n* = 10,111). Ultimately, 146,832 individuals with dementia (52,124 men and 94,708 women) were included in the analyses.

### Ethical approval

This study complied with the provisions of the Declaration of Helsinki and was approved by the Institutional Review Board of the Korea University Guro Hospital, Seoul, Korea (No. 2022GR0325). The requirement for written informed consent was waived since all data used in the analysis were anonymous and non-identifiable.

### Parameters of glycemic status

The participants were divided into normoglycemia and DM groups. Only type 2 DM was selected, which was defined as fasting plasma glucose (FPG) ≥ 126 mg/dL or ≥ 1 anti-diabetic medication prescription per year under ICD-10 codes E11–E14. Glycemic status was categorized into 4 groups as follows: (1) normoglycemia (FPG < 100 mg/dL without a history of claims for anti-diabetic medication and the ICD-10 codes E11–E14), (2) prediabetes (FPG 100–125 mg/dL without claims for anti-diabetic medication and the ICD-10 codes E11–E14), (3) new-onset DM (FPG ≥ 126 mg/dL without previous claims for anti-diabetic medication and the ICD-10 codes E11–E14), and (4) known DM (claims for anti-diabetic medication under the ICD-10 codes E11–E14).

In addition, the duration of diabetes was combined with the glycemic status. Based on this, individuals were divided into five groups as follows: (1) normoglycemia, (2) prediabetes, (3) new-onset DM, (4) DM < 5 years, and (5) DM ≥ 5 years.

### Study outcome and follow-up

The endpoint was all-cause mortality, which was assessed using nationwide death certificate data from the Korea National Statistical Office. The study participants were followed up from one year after the index date until the date of death or until December 31, 2019, whichever came first. The mean follow-up duration was 3.7 ± 2.1 (interquartile range: 2.0 ∼ 5.2) years.

### Covariates

Anthropometric measurements including height, weight, waist circumference, and blood pressure (systolic and diastolic) were measured by healthcare professionals. Body mass index (BMI) was calculated as weight divided by height in meters squared (kg/m^2^). Blood samples were collected after overnight fasting to measure the concentrations of FPG, total cholesterol, triglycerides, high-density lipoprotein (HDL) cholesterol, low-density lipoprotein (LDL) cholesterol, and creatinine.

Comorbidities were defined on the basis of a combination of health examination results and claims for medication prescriptions before the index date. Hypertension was defined as a systolic/diastolic blood pressure of ≥ 140/90 mmHg or ≥ 1 medication prescription per year under ICD-10 codes I10–I13 or I15. Dyslipidemia was defined as a total cholesterol concentration of ≥ 240 mg/dL or ≥ 1 medication prescription per year under ICD-10 code E78. Chronic kidney disease (CKD) was defined as estimated glomerular filtration rate < 60 mL/minute/1.73 m^2^ calculated using the modification of diet in renal disease equation.

Data on smoking status, alcohol consumption, and physical activity were collected using self-reported questionnaires. Smoking status was divided into two categories: ever smokers and never smokers. Alcohol drinkers were defined as individuals who consumed ≥ 1 g of average alcohol per day. Regular exercise was defined as vigorous exercise for ≥ 3 days per week or moderate exercise for ≥ 5 days per week.

Low income was defined as individuals at the lowest 25th percentile using the NHI premium as a proxy for income level, and eligible for medical aid. Place of residence was divided into two groups: urban (metropolitan and city) and rural areas.

Disability was defined based on national disability registration data, with a focus on disabilities resulting from brain impairment. This definition was used to assess the severity of dementia [18]. The number of anti-dementia medications was categorized into 1 to 4 according to the prescription within one year of dementia diagnosis.

### Statistical analyses

Baseline characteristics according to glycemic status were presented as means ± standard deviation (SD) for continuous variables or numbers (percentages) for categorical variables. Continuous variables were compared using analysis of variance, and categorical variables were compared using the chi-squared test. The mortality rate was calculated by dividing the number of deaths by 1,000 person-years.

Survival probabilities according to glycemic status parameters were plotted using Kaplan-Meier curves and compared using log-rank tests. We performed multivariable Cox proportional hazards regression analyses to evaluate the associations between glycemic status parameters and the risk of all-cause mortality, and the results were reported as hazard ratios (HRs) and 95% confidence intervals (CIs). Three models were used: Model 1 was not adjusted for any variables; Model 2 was adjusted for age, sex, place of residence, income, smoking status, alcohol consumption, physical activity, BMI, hypertension, dyslipidemia, and CKD; and Model 3 was adjusted for disability and the number of anti-dementia medications in addition to the variables in Model 2. We performed a sensitivity analysis excluding participants who experienced all-cause mortality within 2 years of follow-up. Subgroup analyses were performed after stratified by all confounding variables.

All statistical analyses were performed using the SAS software (version 9.4; SAS Institute, Cary, NC, USA). Differences were considered statistically significant at *P* < 0.05.

### Data and resource availability

Restrictions apply to the availability of all data analyzed in this study because they were used under license. Additional data are available through approval and oversight by the Korean NHIS.

## Results

### Baseline characteristics of study population

Table 1 shows the baseline characteristics of 146,832 individuals with dementia according to their glycemic status. The proportions of Alzheimer’s disease and vascular dementia were 70.5% and 13.3%, respectively. Among them, 67,771 (46.2%) were found to be categorized into normoglycemia, 35,365 (24.1%) into prediabetes, 8,066 (5.5%) into new-onset DM, 9,247 (6.3%) into DM < 5 years, and 26,383 (17.9%) into DM ≥ 5 years, respectively.

**Table 1:** Baseline characteristics of study population

Variables	Normoglycemia (*n* = 67,771)	Prediabetes (*n* = 35,365)	New-onset DM (*n* = 8,066)	DM < 5 years (*n* = 9,247)	DM ≥ 5 years (*n* = 26,383)	*P*-value
Age (years)	75.1 ± 9.7	75.4 ± 9.1	77.0 ± 9.3	73.2 ± 8.8	74.9 ± 7.6	< 0.001
Sex (men)	23 563 (34.8)	12,625 (35.7)	2,840 (35.2)	3,694 (40.0)	9,402 (35.6)	< 0.001
Urban residence	20,383 (30.1)	11,236 (31.8)	2,478 (30.7)	3,132 (33.9)	8,790 (33.3)	< 0.001
Low income	15,303 (22.6)	7,786 (22.0)	1,941 (24.1)	2,209 (23.9)	5,746 (21.8)	< 0.001
Ever smoker	12,769 (18.8)	6,975 (19.7)	1,472 (18.3)	2,197 (23.8)	5,188 (19.7)	< 0.001
Alcohol drinker	5,831 (8.6)	3,702 (10.5)	793 (9.8)	891 (9.6)	2,051 (7.8)	< 0.001
Regular exerciser	6,355 (9.4)	3,430 (9.7)	514 (6.4)	908 (9.8)	2,582 (9.8)	< 0.001
Body mass index (kg/m^2^)	22.6 ± 3.5	23.2 ± 3.6	23.0 ± 3.8	23.9 ± 3.6	23.6 ± 3.5	< 0.001
Waist circumference (cm)	80.4 ± 9.4	82.1 ± 9.5	82.0 ± 10.2	84.1 ± 9.5	84.1 ± 9.4	< 0.001
Systolic BP (mmHg)	126.1 ± 16.4	127.7 ± 16.5	127.3 ± 16.8	127.7 ± 16.3	128.5 ± 16.8	< 0.001
Diastolic BP (mmHg)	76.1 ± 10.4	76.9 ± 10.4	76.8 ± 10.5	76.8 ± 10.3	75.6 ± 10.3	< 0.001
FPG (mg/dL)	88.3 ± 7.8	108.9 ± 7.0	152.9 ± 39.7	119.2 ± 40.2	137.8 ± 57.5	< 0.001
Total cholesterol (mg/dL)	188.0 ± 40.7	193.5 ± 42.7	192.5 ± 44.7	180.9 ± 43.1	175.4 ± 43.1	< 0.001
Triglycerides (mg/dL)	128.1 ± 66.5	137.0 ± 73.8	152.4 ± 89.4	149.4 ± 82.0	148.8 ± 82.4	< 0.001
HDL-cholesterol (mg/dL)	51.6 ± 15.4	51.8 ± 14.8	49.5 ± 14.5	49.3 ± 14.5	48.1 ± 14.7	< 0.001
LDL-cholesterol (mg/dL)	110.8 ± 36.2	114.3 ± 38.2	112.9 ± 39.7	102.0 ± 37.8	97.8 ± 37.8	< 0.001
eGFR (mL/min/1.73m^2^)	79.8 ± 35.0	77.8 ± 38.6	76.4 ± 46.1	78.4 ± 41.6	70.8 ± 37.5	< 0.001
Obesity	16,130 (23.8)	10,563 (29.9)	2,311 (28.7)	3,373 (36.6)	8,537 (32.4)	< 0.001
Hypertension	41,841 (61.7)	23,821 (67.4)	5,303 (65.8)	7,304 (79.0)	21,821 (82.7)	< 0.001
Dyslipidemia	22,874 (33.8)	13,914 (39.3)	2,887 (35.8)	5,230 (56.6)	15,155 (57.4)	< 0.001
Chronic kidney disease	12,978 (19.2)	7,836 (22.2)	2,166 (26.9)	2,221 (24.0)	9,512 (36.1)	< 0.001
Subtype of dementia						< 0.001
Alzheimer’s disease	47,785 (70.5)	25,194 (71.2)	5,731 (71.1)	6,227 (67.3)	18,507 (70.2)	
Vascular dementia	8,937 (13.2)	4,478 (12.7)	1,001 (12.4)	1,475 (16.0)	3,637 (13.8)	
Others	11,049 (16.3)	5,693 (16.1)	1,334 (16.5)	1,545 (16.7)	4,239 (16.1)	
Number of anti-dementia medication						0.228
1	58,132 (85.8)	30,371 (85.9)	6,841 (84.8)	7,909 (85.5)	22,663 (85.9)	
2	8,867 (13.1)	4,592 (13.0)	1,139 (14.1)	1,215 (13.1)	3,408 (12.9)	
≥ 3	772 (1.1)	402 (1.1)	86 (1.1)	123 (1.3)	312 (1.2)	

Abbreviation: DM, diabetes mellitus; BP, blood pressure; FPG, fasting plasma glucose; HDL, high-density lipoprotein; LDL, low-density lipoprotein; eGFR, estimated glomerular filtration rate.

Values are presented as means ± standard deviations or numbers (percentages)

The mean age of the study subjects was 75.1 ± 9.2 years, with 87.1% being 65 years or older, and 35.5% being men. Individuals who had been diagnosed with DM within 5 years had the highest proportion of men, urban residents, ever-smokers, and the highest BMI and waist circumference. Individuals who have suffered from DM ≥ 5 years had the highest systolic blood pressure and the lowest eGFR values and had the higher proportions of comorbidities such as hypertension, dyslipidemia, and CKD than other groups.

### Associations between glycemic status and all-cause mortality in individuals with dementia

**Fig. 1:**
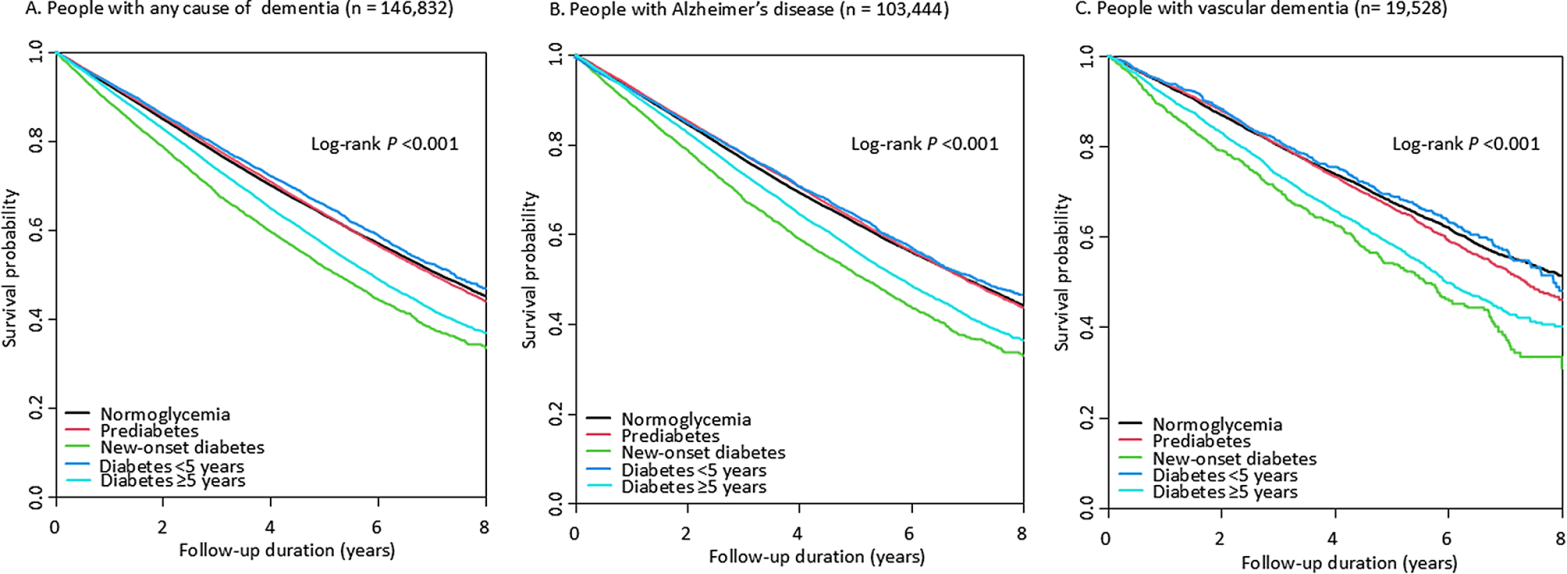
Kaplan-Meier curves for survival probability according to the glycemic status in individuals with dementia (**A**) All cause-dementia, (**B**) Alzheimer’s disease, and (**C**) Vascular dementia

During the mean follow-up of 3.7 ± 2.1 years, there were 52,118 (35.2%) deaths in all-cause dementia, 36,988 (35.8%) deaths in Alzheimer’s disease, and 6,236 (31.9%) deaths in vascular dementia, respectively. The Kaplan-Meier curves in Fig. 1 show significantly lower survival probabilities among individuals with dementia, new-onset DM, and DM with a longer duration (log-rank test *P* < 0.001).

Table 2 presents the longitudinal associations between glycemic status and the risk of all-cause mortality in individuals with dementia. Among individuals with all-cause dementia, those with DM were associated with an increased risk of all-cause mortality, compared to those without DM, after adjusting for confounding variables (Model 3, HR 1.34, 95% CI: 1.32–1.37). Compared with normoglycemia group, the risk of all-cause mortality was the highest in known DM (HR 1.36, 95% CI: 1.33–1.39) and new-onset DM (HR 1.35, 95% CI: 1.30–1.40), and followed by prediabetes (HR 1.03, 95% CI: 1.01–1.05). These associations persisted in both Alzheimer’s disease and vascular dementia, although the association between prediabetes and mortality risk was attenuated in individuals with vascular dementia.

**Table 2:** Associations between glycemic status and risk of all-cause mortality in individuals with dementia

Group		Dementia (*n*)	Mortality (*n*)	Person-years	Mortality rate^*^	HR (95% CI)^†^		
						Model 1^‡^	Model 2^§^	Model 3^||^
**All-cause dementia**								
DM	No	103,136	35,309	388,769	90.8	1 (reference)	1 (reference)	1 (reference)
	Yes	43,696	16,809	155,428	108.2	1.20 (1.18–1.22)	1.35 (1.32–1.37)	1.34 (1.32–1.37)
Glycemic status	Normoglycemia	67,771	23,386	256,459	91.2	1 (reference)	1 (reference)	1(reference)
	Prediabetes	35,365	11,923	132,310	90.1	0.99 (0.97–1.01)	1.03 (1.00–1.05)	1.03 (1.01–1.05)
	New-onset DM	8,066	3,545	27,215	130.3	1.45 (1.40–1.50)	1.35 (1.30–1.40)	1.35 (1.30–1.40)
	Known DM	35,630	13,264	128,213	103.5	1.14 (1.12–1.17)	1.36 (1.33–1.39)	1.36 (1.33–1.39)
Glycemic status and duration of DM	Normoglycemia	67,771	23,386	256,459	91.2	1 (reference)	1 (reference)	1 (reference)
	Prediabetes	35,365	11,923	132,310	90.1	0.99 (0.97–1.01)	1.03 (1.01–1.05)	1.03 (1.01–1.05)
	New-onset DM	8,066	3,545	27,215	130.3	1.45 (1.40–1.50)	1.35 (1.30–1.40)	1.35 (1.30–1.40)
	DM < 5 years	9,247	3,104	36,231	85.7	0.94 (0.90–0.97)	1.17 (1.13–1.21)	1.17 (1.13–1.21)
	DM ≥ 5 years	26,383	10,160	91,983	110.5	1.23 (1.20–1.26)	1.43 (1.40–1.47)	1.43 (1.40–1.47)
**Alzheimer’s disease**								
DM	No	72,979	25,247	271,812	92.9	1 (reference)	1 (reference)	1 (reference)
	Yes	30,465	11,741	107,012	109.7	1.19 (1.17–1.22)	1.35 (1.32–1.38)	1.34 (1.31–1.37)
Glycemic status	Normoglycemia	47,785	16,721	178,674	93.6	1 (reference)	1 (reference)	1 (reference)
	Prediabetes	25,194	8,526	93,138	91.5	0.98 (0.96–1.01)	1.03 (1.00–1.05)	1.03 (1.00–1.05)
	New-onset DM	5,731	2,527	19,154	131.9	1.43 (1.37–1.49)	1.34 (1.29–1.40)	1.34 (1.29–1.40)
	Known DM	24,734	9,214	87,857	104.9	1.13 (1.10–1.16)	1.36 (1.33–1.40)	1.36 (1.32–1.40)
Glycemic status and duration of DM	Normoglycemia	47,785	16,721	178,674	93.6	1 (reference)	1 (reference)	1 (reference)
	Prediabetes	25,194	8,526	93,138	91.5	0.98 (0.96–1.01)	1.03 (1.00–1.05)	1.03 (1.00–1.05)
	New-onset DM	5,731	2,527	19,154	131.9	1.43 (1.37–1.49)	1.34 (1.29–1.40)	1.34 (1.29–1.40)
	DM < 5 years	6,227	2,117	24,145	87.7	0.93 (0.89–0.98)	1.18 (1.13–1.23)	1.18 (1.13–1.23)
	DM ≥ 5 years	18,507	7,097	63,713	111.4	1.21 (1.17–1.24)	1.43 (1.39–1.47)	1.43 (1.39–1.47)
**Vascular dementia**								
DM	No	13,415	4,036	50,991	79.2	1 (reference)	1 (reference)	1 (reference)
	Yes	6,113	2,200	21,774	101.0	1.29 (1.22–1.36)	1.40 (1.33–1.48)	1.39 (1.32–1.47)
Glycemic status	Normoglycemia	8,937	2,668	34,224	78.0	1 (reference)	1 (reference)	1 (reference)
	Prediabetes	4,478	1,368	16,767	81.6	1.05 (1.00–1.12)	1.06 (1.00–1.13)	1.06 (0.99–1.13)
	New-onset DM	1,001	411	3,312	124.1	1.62 (1.46–1.80)	1.45 (1.31–1.61)	1.45 (1.30–1.61)
	Known DM	5,112	1,789	18,461	96.9	1.25 (1.18–1.33)	1.42 (1.33–1.51)	1.41 (1.33–1.50)
Glycemic status and duration of DM	Normoglycemia	8,937	2,668	34,224	78.0	1 (reference)	1 (reference)	1 (reference)
	Prediabetes	4,478	1,368	16,767	81.6	1.05 (0.99–1.12)	1.06 (0.99–1.13)	1.06 (0.99–1.13)
	New-onset DM	1,001	411	3,312	124.1	1.62 (1.46–1.80)	1.45 (1.31–1.61)	1.45 (1.31–1.61)
	DM < 5 years	1,475	435	5,782	75.2	0.96 (0.87–1.06)	1.17 (1.05–1.29)	1.16 (1.05–1.29)
	DM ≥ 5 years	3,637	1,354	12,680	106.8	1.39 (1.30–1.48)	1.52 (1.42–1.63)	1.52 (1.42–1.63)

Abbreviations: HR, hazard ratio; CI, confidence interval; DM, diabetes mellitus.

^*^Mortality per 1000 person-years. ^†^HRs (95% CIs) were calculated using a multivariable Cox hazards regression analysis.

^‡^Model 1 was not adjusted for any variables. ^§^Model 2 was adjusted for age, sex, place of residence, income, smoking status, alcohol consumption, physical activity, body mass index, hypertension, dyslipidemia, and chronic kidney disease. ^||^Model 3 was adjusted for age, sex, place of residence, income, smoking status, alcohol consumption, physical activity, body mass index, hypertension, dyslipidemia, chronic kidney disease, disability, and number of anti-dementia medication

When the duration of DM was considered, the HR for all-cause mortality was higher in people with longer duration of DM than those with shorter duration of DM: HR 1.43 (95% CI: 1.40–1.47) for those with DM ≥ 5 years vs. HR 1.17 (95% CI: 1.13–1.21) for those with DM < 5 years. These associations persisted in individuals with Alzheimer’s disease and vascular dementia. Furthermore, the sensitivity analysis excluding participants who died within 2 years of follow-up were performed (Supplementary Table S1). The observed associations remained unchanged.

### Subgroup analyses

Table 3 show the association between glycemic status and the risk of all-cause mortality, after stratified by age and sex. Overall, similar trends were observed in all the subgroups. There were significant interactions with age (*P* for interaction < 0.001) in the association between glycemic status and all-cause mortality in individuals with all-cause dementia and Alzheimer’s disease. This association was prominent in younger adults (40–64 years old) with all-cause dementia and Alzheimer’s disease. Among individuals with vascular dementia, this association was more prominent in men than in women (*P* for interaction = 0.041). Additionally, among individuals with all-cause dementia, the association between glycemic status and mortality was more pronounced in drinkers and those with obesity, hypertension, dyslipidemia, and CKD, compared to nondrinkers and those without these comorbidities (Supplementary Table S2). In individuals with Alzheimer’s disease, this association was also more pronounced in those with obesity, dyslipidemia, CKD, and those taking fewer than two anti-dementia medications, compared to those without these conditions (Supplementary Table S3). Among individuals with vascular dementia, the association was more prominent in those with low income, hypertension, dyslipidemia, and those taking fewer than two anti-dementia medications, compared to those without these conditions (Supplementary Table S4).

**Table 3:** Subgroup analysis for the associations between glycemic status and risk of all-cause mortality in individuals with dementia

Subgroup	Normoglycemia	Prediabetes^*^	New-onset DM^*^	DM < 5 years^*^	DM ≥ 5 years^*^	*P* for interaction
**All-cause dementia**						
Age (years)						< 0.001
40–64	1 (reference)	1.13 (1.02–1.25)	1.96 (1.67–2.30)	1.45 (1.27–1.66)	1.77 (1.60–1.96)	
≥ 65	1 (reference)	1.02 (1.00–1.05)	1.32 (1.28–1.37)	1.15 (1.11–1.20)	1.42 (1.39–1.46)	
Sex						0.052
Men	1 (reference)	1.02 (0.98–1.05)	1.28 (1.21–1.36)	1.20 (1.14–1.27)	1.41 (1.36–1.46)	
Women	1 (reference)	1.04 (1.01–1.07)	1.39 (1.33–1.46)	1.14 (1.08–1.20)	1.45 (1.40–1.50)	
**Alzheimer’s disease**						
Age (years)						< 0.001
40–64	1 (reference)	1.13 (0.99–1.29)	2.03 (1.66–2.49)	1.56 (1.31–1.86)	1.79 (1.57–2.04)	
≥ 65	1 (reference)	1.02 (1.00–1.05)	1.32 (1.26–1.38)	1.16 (1.11–1.22)	1.42 (1.38–1.46)	
Sex						0.172
Men	1 (reference)	1.00 (0.96–1.04)	1.29 (1.20–1.38)	1.19 (1.11–1.27)	1.38 (1.32–1.45)	
Women	1 (reference)	1.05 (1.01–1.08)	1.37 (1.30–1.45)	1.17 (1.10–1.24)	1.46 (1.40–1.51)	
**Vascular dementia**						
Age (years)						0.150
40–64	1 (reference)	1.04 (0.82–1.30)	1.81 (1.24–2.63)	1.33 (0.99–1.78)	1.91 (1.54–2.37)	
≥ 65	1 (reference)	1.06 (0.99–1.13)	1.42 (1.28–1.59)	1.15 (1.03–1.28)	1.49 (1.39–1.60)	
Sex						0.041
Men	1 (reference)	1.14 (1.03–1.25)	1.31 (1.12–1.53)	1.22 (1.06–1.40)	1.55 (1.41–1.71)	
Women	1 (reference)	0.99 (0.90–1.09)	1.59 (1.38–1.82)	1.11 (0.95–1.29)	1.49 (1.36–1.63)	

Abbreviations: HR, hazard ratio; CI, confidence interval; DM, diabetes mellitus.

^*^HRs (95% CIs) were calculated using a multivariable Cox hazards regression model adjusted for age, sex, place of residence, income, smoking status, alcohol consumption, physical activity, body mass index, hypertension, dyslipidemia, chronic kidney disease, disability, and number of anti-dementia medication

## Discussion

In this nationwide, large-scale cohort of people with dementia, we demonstrated an association between glycemic dysregulation and the risk of all-cause mortality. The presence of DM was significantly associated with a 1.34-fold increased risk of mortality compared to the absence of DM. Prediabetes was also associated with a slightly but significantly increased risk of mortality compared with normoglycemia. Regarding the status of glucose metabolism, the mortality risk in people with new-onset DM and DM with a longer duration was higher than that in those with DM < 5 years. These associations were maintained in both Alzheimer’s disease and vascular dementia. In stratified analyses, these associations were stronger in younger individuals with all-cause dementia and Alzheimer’s disease than in elderly individuals, and in men with vascular dementia than in women.

Several studies reported that comorbid DM is associated with poor prognosis and higher death in people with dementia [6, 19, 20]. A study including 25,006 individuals with dementia showed that those with history of DM died 2.6 (95% CI: 2.3–2.9) years earlier than those without history of DM [18]. In particular, individuals who suffered DM ≥ 15 years died 6.9 (4.4–9.5) years earlier than those without DM among the patients with vascular dementia [19]. Another study of 323 patients with Alzheimer’s disease reported that individuals with preexisting DM had a 1.99-fold (95% CI: 1.32–2.99) increased risk of shorter lifespan compared with those without [20]. In a meta-analysis including nine studies with 13,000 patients with dementia, those with preexisting DM had an increased mortality risk compared to those without (HR 1.49, 95% CI: 1.33–1.68) [6].

In contrast, it was reported that a history of DM was not associated with 5-year all-cause mortality in 54 European descendants with dementia [14]. Another study showed that previous DM status was not significantly associated with all-cause mortality within 6 months in 123 Americans with advanced dementia [21]. However, that study recruited nursing home residents with end-stage dementia. Thus, the association between the glycemic regulation status and mortality in people with dementia has not yet been determined. Our study confirmed that DM comorbidity is clearly associated with increased mortality in people with dementia, and provided additional findings that a longer duration of DM was associated with an increased risk of all-cause mortality in this population.

There are several possible mechanisms through which DM affects the clinical course of patients with dementia. First, the pathophysiology of DM is grounded in insulin resistance and is associated with chronic inflammation, endothelial dysfunction, and oxidative stress [22]. In addition, DM is associated with an increased risk of microvascular and macrovascular complications, which can accelerate the clinical outcomes in patients with dementia [23, 24]. Of note, DM is also related to Alzheimer’s disease-related changes, including ß-amyloid deposits [25]. In this context, people with DM, particularly those with a longer duration, are more likely to deteriorate the clinical expression of dementia. Several studies have also reported that DM is associated with unfavorable changes in white matter microstructure and an increased risk of microbleeding [26, 27].

Notably, we found an increased risk of all-cause mortality in new-onset DM, which was nearly equal to that in known DM and long-standing DM (≥ 5 years of duration). To our knowledge, there was no evidence on the association between new-onset DM (vs. DM with short duration) and mortality among people with dementia. A cohort study in South Korea showed that the risk of dementia was higher in individuals with new-onset DM compared to those with shorter duration of DM [8]. These data along with ours suggest that some people with new-onset DM are likely to be in not well controlled in glucose regulation such as hyperosmolar hyperglycemic status, leading to increased mortality. It was reported that high glycemic status was linked to accelerated cognitive decline and microvascular lesions in cerebrovascular system [28]. A recent study reported that hyperglycemia in patients with acute coronary syndrome was associated with cardiovascular mortality [29]. Complex mechanisms such as acute glucotoxicity, inflammatory cytokines, oxidative stress, and electrolyte imbalance, and hormonal derangements are involved in this phenomenon [30–32]. On the contrary, people who surpassed acute glycemic status may have stable glucose regulation status. In addition, individuals newly diagnosed with DM at an older age, might have a poorer functional status, lower accessibility to medical services, or a lack of long-term health checkups, which may lead to a poor prognosis, although our analyses could not fully address these issues.

Furthermore, prediabetes was also associated with increased mortality in people with dementia and Alzheimer’s disease. Glucose dysregulation has a wide spectrum from prediabetes, such as impaired fasting glucose or impaired glucose tolerance to DM, with unfavorable metabolic changes starting from prediabetes [8]. Therefore, maintaining normal blood glucose levels might be critical for reducing the risk of all-cause mortality among individuals with dementia. Meanwhile, the American Diabetes Association recommended that the treatment goal of glycemia in relatively healthy older adults is hemoglobin A1c 7.0–7.5%, while the treatment goals in elderly adults with comorbidities or frailty is less stringent in type 2 diabetes [33]. Further studies are needed to evaluate the target of glycemic control to reduce the risk of all-cause mortality in patients with dementia.

In our study, the risk of all-cause mortality was the highest among DM ≥ 5 years, followed by new onset DM, and DM < 5 years, even adjusting for important variables including age and sex. These associations were more pronounced in the younger adults with all-cause dementia and Alzheimer’s disease. Our findings were supported by a prior study that demonstrated that associations between history of DM and mortality were stronger among younger adults than elderly adults in patients with dementia [19]. This is mainly likely to come from the fact that the disease duration is longer when it starts at young age. In addition, the significant association between glycemic status and all-cause mortality was more pronounced among men with vascular dementia than among women. Therefore, glycemic control might be more critical in men to reduce the risk of all-cause mortality, particularly because dementia of vascular origin originates from atherosclerosis and vascular dysfunction, which are commonly found in DM [34, 35]. In addition, these associations were more pronounced in individuals with comorbidities compared to those without. Therefore, maintaining good glycemic control may be crucial for reducing the risk of all-cause mortality among patients with dementia, particularly those with additional comorbidities.

The present study has several limitations. First, there might be reverse causality between glycemic status and mortality because of the retrospective design, although we considered a 1-year of lag time. Second, there were residual confounding factors, such as social support, educational level [36], and genetic factors (e.g., apolipoprotein E4), that could have affected all-cause mortality among individuals with dementia because they were not measured in the NHIS database. Third, our database does not include specific causes of mortality. Fourth, as the Korean NHIS health examination data include only FPG levels for assessing glycemic status, we could not evaluate glycemic variability indices. Finally, this study was conducted only in an Asian population.

Despite these limitations, our study has several strengths. We investigated the risk of all-cause mortality among individuals with dementia using large-scale data representing entire country. This enabled us to conduct comprehensive adjustments for various confounding variables and perform subgroup analyses. We used fasting glucose data to define the glycemic regulation status, and not based on an individual’s report. To our knowledge, this is the first study to investigate the association between DM duration and mortality risk in people with dementia.

In conclusion, we found that glycemic dysregulation, including prediabetes, was associated with an increased risk of all-cause mortality among individuals with dementia. Furthermore, individuals with new-onset DM and a longer duration of DM showed an increased risk of all-cause mortality compared to those with a shorter duration of diabetes. Based on these findings, maintaining normal glucose levels may reduce the risk of all-cause mortality in individuals with dementia.

### Electronic supplementary material

Below is the link to the electronic supplementary material.

Supplementary Material 1

## Data Availability

Additional data are available through approval and oversight by the Korean National Health Insurance Service.
