# Supplementary material for: Association between glycemic status and all-cause mortality among individuals with dementia: a nationwide cohort study
Source: Alzheimers Res Ther. 2024 Aug 22;16:191. doi: 10.1186/s13195-024-01557-x (PMC11340194; doi:10.1186/s13195-024-01557-x)
Supplement: Supplementary file 1 — Supplementary Material 1 [file 13195_2024_1557_MOESM1_ESM.docx]

| Supplemental Table S1. Sensitivity analysis excluding participants who died within 2 years of follow-up | | | | | | | | |
| --- | --- | --- | --- | --- | --- | --- | --- | --- |
| Group | | Dementia (n) | Mortality (n) | Person-years | Mortality rate^*^ | HR (95% CI)^†^ |  |  |
|  |  |  |  |  |  | Model 1^‡^ | Model 2^§^ | Model 3^\|\|^ |
| **All-cause dementia** | | | | | | | | |
| DM | No | 95,549 | 27,723 | 289,384 | 95.8 | 1 (reference) | 1 (reference) | 1 (reference) |
|  | Yes | 39,887 | 13,000 | 113,628 | 114.4 | 1.20 (1.18–1.23) | 1.33 (1.31–1.36) | 1.33 (1.30–1.36) |
| Glycemic status | Normoglycemia | 62,683 | 18,298 | 191,213 | 95.7 | 1 (reference) | 1 (reference) | 1 (reference) |
|  | Prediabetes | 32,866 | 9,425 | 98,172 | 96.0 | 1.01 (0.98–1.03) | 1.03 (1.01–1.06) | 1.03 (1.01–1.06) |
|  | New-onset DM | 7,152 | 2,631 | 19,612 | 134.2 | 1.42 (1.36–1.48) | 1.33 (1.27–1.38) | 1.32 (1.27–1.38) |
|  | Known DM | 32,735 | 10,369 | 94,016 | 110.3 | 1.16 (1.13–1.19) | 1.36 (1.32–1.39) | 1.35 (1.32–1.39) |
| Glycemic status and duration of DM | Normoglycemia | 62,683 | 18,298 | 191,213 | 95.7 | 1 (reference) | 1 (reference) | 1 (reference) |
|  | Prediabetes | 32,866 | 9,425 | 98,172 | 96.0 | 1.01 (0.98–1.03) | 1.03 (1.01–1.06) | 1.03 (1.01–1.06) |
|  | New-onset DM | 7,152 | 2,631 | 19,612 | 134.2 | 1.42 (1.36–1.48) | 1.33 (1.27–1.38) | 1.32 (1.27–1.38) |
|  | DM <5 years | 8,607 | 2,464 | 27,316 | 90.2 | 0.94 (0.90–0.98) | 1.15 (1.10–1.20) | 1.15 (1.10–1.20) |
|  | DM ≥5 years | 24,128 | 7,905 | 66,700 | 118.5 | 1.25 (1.22–1.29) | 1.44 (1.40–1.48) | 1.43 (1.40–1.47) |
| **Alzheimer’s disease** | | | | | | | | |
| DM | No | 67,455 | 19,724 | 201,569 | 97.9 | 1 (reference) | 1 (reference) | 1 (reference) |
|  | Yes | 27,807 | 9,083 | 77,872 | 116.6 | 1.20 (1.17–1.23) | 1.34 (1.31–1.37) | 1.34 (1.30–1.37) |
| Glycemic status | Normoglycemia | 44,091 | 13,027 | 132,720 | 98.2 | 1 (reference) | 1 (reference) | 1 (reference) |
|  | Prediabetes | 23,364 | 6,697 | 68,849 | 97.3 | 0.99 (0.97–1.02) | 1.03 (1.00–1.06) | 1.03 (1.00–1.06) |
|  | New-onset DM | 5,094 | 1,890 | 13,750 | 137.5 | 1.42 (1.35–1.49) | 1.34 (1.27–1.40) | 1.34 (1.27–1.40) |
|  | Known DM | 22,713 | 7,193 | 64,123 | 112.2 | 1.15 (1.12–1.19) | 1.3 6(1.32–1.40) | 1.36 (1.31–1.40) |
| Glycemic status and duration of DM | Normoglycemia | 44,091 | 13,027 | 132,720 | 98.2 | 1 (reference) | 1 (reference) | 1 (reference) |
|  | Prediabetes | 23,364 | 6,697 | 68,849 | 97.3 | 0.99 (0.97–1.02) | 1.03 (1.00–1.06) | 1.03 (1.00–1.06) |
|  | New-onset DM | 5,094 | 1,890 | 13,750 | 137.5 | 1.42 (1.35–1.49) | 1.34 (1.28–1.41) | 1.34 (1.27–1.40) |
|  | DM <5 years | 5,783 | 1,673 | 18,148 | 92.2 | 0.94 (0.89–0.99) | 1.153(1.10–1.21) | 1.15 (1.10–1.21) |
|  | DM ≥5 years | 16,930 | 5,520 | 45,975 | 120.1 | 1.24 (1.20–1.28) | 1.43 (1.39–1.48) | 1.43 (1.39–1.48) |
| **Vascular dementia** | | | | | | | | |
| DM | No | 12,583 | 3,204 | 37,981 | 84.4 | 1 (reference) | 1 (reference) | 1 (reference) |
|  | Yes | 5,593 | 1,680 | 15,909 | 105.6 | 1.26 (1.19–1.34) | 1.36 (1.28–1.45) | 1.36 (1.28–1.44) |
| Glycemic status | Normoglycemia | 8,371 | 2,102 | 25,562 | 82.2 | 1 (reference) | 1 (reference) | 1 (reference) |
|  | Prediabetes | 4,212 | 1,102 | 12,419 | 88.7 | 1.08 (1.01–1.17) | 1.07 (1.00–1.15) | 1.07 (1.00–1.16) |
|  | New-onset DM | 885 | 295 | 2,367 | 124.6 | 1.54 (1.36–1.74) | 1.38 (1.23–1.56) | 1.38 (1.22–1.56) |
|  | Known DM | 4,708 | 1,385 | 13,542 | 102.3 | 1.25 (1.17–1.34) | 1.40 (1.30–1.50) | 1.39 (1.30–1.50) |
| Glycemic status and duration of DM | Normoglycemia | 8,371 | 2,102 | 25,562 | 82.2 | 1 (reference) | 1 (reference) | 1 (reference) |
|  | Prediabetes | 4,212 | 1,102 | 12,419 | 88.7 | 1.08 (1.01–1.17) | 1.07 (1.00–1.15) | 1.07 (1.00–1.16) |
|  | New-onset DM | 885 | 295 | 2,367 | 124.6 | 1.54 (1.36–1.74) | 1.38 (1.23–1.57) | 1.38 (1.22–1.56) |
|  | DM <5 years | 1,388 | 348 | 4,349 | 80.0 | 0.97 (0.87–1.09) | 1.15 (1.02–1.29) | 1.14 (1.02–1.28) |
|  | DM ≥5 years | 3,320 | 1,037 | 9,193 | 112.8 | 1.39 (1.29–1.50) | 1.51 (1.40–1.63) | 1.50 (1.39–1.62) |
| Abbreviations: HR, hazard ratio; CI, confidence interval; DM, diabetes mellitus.  ^*^Mortality per 1000 person-years. ^†^HRs (95% CIs) were calculated using a multivariable Cox hazards regression analysis.  ^‡^Model 1 was not adjusted for any variables. ^§^Model 2 was adjusted for age, sex, place of residence, income, smoking status, alcohol consumption, physical activity, body mass index, hypertension, dyslipidemia, and chronic kidney disease. ^\|\|^Model 3 was adjusted for age, sex, place of residence, income, smoking status, alcohol consumption, physical activity, body mass index, hypertension, dyslipidemia, chronic kidney disease, disability, and number of anti-dementia medication. | | | | | | | | |

Supplemental Table S2. Additional subgroup analysis in individuals with all-cause dementia

| Subgroup | Normoglycemia | Prediabetes^*^ | New-onset DM^*^ | DM <5 years^*^ | DM ≥5 years^*^ | *P* for  interaction |
| --- | --- | --- | --- | --- | --- | --- |
| Place of residence |  |  |  |  |  | 0.537 |
| Urban | 1 (reference) | 1.05 (1.01–1.09) | 1.35 (1.26–1.44) | 1.14 (1.07–1.22) | 1.45 (1.39–1.51) |  |
| Rural | 1 (reference) | 1.02 (0.99–1.05) | 1.35 (1.29–1.41) | 1.18 (1.13–1.24) | 1.42 (1.38–1.46) |  |
| Income |  |  |  |  |  | 0.339 |
| Others | 1 (reference) | 1.04 (1.01–1.06) | 1.34 (1.29–1.40) | 1.18 (1.13–1.23) | 1.43 (1.39–1.46) |  |
| Low | 1 (reference) | 1.00 (0.95–1.05) | 1.37 (1.27–1.47) | 1.14 (1.05–1.23) | 1.46 (1.38–1.53) |  |
| Smoking status |  |  |  |  |  | 0.424 |
| Never | 1 (reference) | 1.03 (1.01–1.06) | 1.35 (1.30–1.40) | 1.16 (1.11–1.21) | 1.42 (1.38–1.46) |  |
| Ever | 1 (reference) | 1.01 (0.96–1.06) | 1.34 (1.23–1.45) | 1.21 (1.12–1.30) | 1.47 (1.40–1.55) |  |
| Alcohol consumption |  |  |  |  |  | 0.008 |
| Nondrinker | 1 (reference) | 1.02 (1.00–1.05) | 1.34 (1.29–1.39) | 1.15 (1.10–1.19) | 1.42 (1.39–1.46) |  |
| Drinker | 1 (reference) | 1.08 (1.00–1.17) | 1.47 (1.30–1.66) | 1.43 (1.26–1.62) | 1.58 (1.44–1.72) |  |
| Physical activity |  |  |  |  |  | 0.466 |
| Irregular | 1 (reference) | 1.02 (1.00–1.05) | 1.35 (1.30–1.40) | 1.17 (1.13–1.22) | 1.43 (1.40–1.47) |  |
| Regular | 1 (reference) | 1.08 (0.99–1.18) | 1.34 (1.14–1.59) | 1.10 (0.95–1.27) | 1.39 (1.27–1.52) |  |
| Body mass index (kg/m^2^) | |  |  |  |  | 0.003 |
| <25 | 1 (reference) | 1.01 (0.99–1.04) | 1.31 (1.26–1.37) | 1.12 (1.07–1.17) | 1.36 (1.32–1.39) |  |
| ≥25 | 1 (reference) | 0.99 (0.94–1.04) | 1.35 (1.25–1.47) | 1.18 (1.10–1.28) | 1.49 (1.42–1.57) |  |
| Hypertension |  |  |  |  |  | 0.002 |
| No | 1 (reference) | 1.06 (1.02–1.10) | 1.44 (1.36–1.53) | 1.25 (1.15–1.35) | 1.38 (1.31–1.46) |  |
| Yes | 1 (reference) | 1.01 (0.98–1.04) | 1.30 (1.25–1.36) | 1.14 (1.10–1.19) | 1.43 (1.40–1.47) |  |
| Dyslipidemia |  |  |  |  |  | 0.005 |
| No | 1 (reference) | 1.02 (0.99–1.05) | 1.35 (1.29–1.40) | 1.19 (1.13–1.25) | 1.38 (1.34–1.43) |  |
| Yes | 1 (reference) | 1.05 (1.01–1.09) | 1.36 (1.27–1.45) | 1.15 (1.09–1.22) | 1.50 (1.45–1.56) |  |
| Chronic kidney disease |  |  |  |  |  | <0.001 |
| No | 1 (reference) | 1.03 (1.01–1.06) | 1.36 (1.30–1.41) | 1.19 (1.14–1.25) | 1.38 (1.34–1.42) |  |
| Yes | 1 (reference) | 1.01 (0.97–1.06) | 1.33 (1.25–1.42) | 1.11 (1.03–1.19) | 1.51 (1.45–1.58) |  |
| Disability |  |  |  |  |  | 0.701 |
| No | 1 (reference) | 1.03 (1.00–1.06) | 1.35 (1.30–1.41) | 1.17 (1.12–1.23) | 1.42 (1.38–1.46) |  |
| Yes | 1 (reference) | 1.02 (0.98–1.07) | 1.34 (1.25–1.43) | 1.15 (1.08–1.24) | 1.46 (1.40–1.52) |  |
| Number of anti-dementia medication | |  |  |  |  | 0.058 |
| <2 | 1 (reference) | 1.03 (1.01–1.06) | 1.37(1.32–1.43) | 1.18 (1.14–1.23) | 1.45 (1.41–1.48) |  |
| ≥2 | 1 (reference) | 1.01 (0.96–1.07) | 1.23 (1.12–1.34) | 1.09 (0.99–1.19) | 1.36 (1.28–1.44) |  |
| Abbreviations: HR, hazard ratio; CI, confidence interval; DM, diabetes mellitus.  ^*^HRs (95% CIs) were calculated using a multivariable Cox hazards regression model adjusted for age, sex, place of residence, income, smoking status, alcohol consumption, physical activity, body mass index, hypertension, dyslipidemia, chronic kidney disease, disability, and number of anti-dementia medication. | | | | | | |

Supplemental Table S3. Additional subgroup analysis in individuals with Alzheimer’s disease

| Subgroup | Normoglycemia | Prediabetes^*^ | New-onset DM^*^ | DM <5 years^*^ | DM ≥5 years^*^ | *P* for  interaction |
| --- | --- | --- | --- | --- | --- | --- |
| Place of residence |  |  |  |  |  | 0.215 |
| Urban | 1 (reference) | 1.06 (1.01–1.11) | 1.30 (1.21–1.41) | 1.13 (1.04–1.22) | 1.43 (1.36–1.50) |  |
| Rural | 1 (reference) | 1.01 (0.98–1.05) | 1.36 (1.29–1.43) | 1.20 (1.14–1.27) | 1.42 (1.38–1.47) |  |
| Income |  |  |  |  |  | 0.987 |
| Others | 1 (reference) | 1.03 (1.00–1.06) | 1.34 (1.28–1.41) | 1.18 (1.12–1.24) | 1.43 (1.39–1.48) |  |
| Low | 1 (reference) | 1.02 (0.97–1.08) | 1.35 (1.24–1.47) | 1.17 (1.06–1.29) | 1.41 (1.32–1.50) |  |
| Smoking status |  |  |  |  |  | 0.475 |
| Never | 1 (reference) | 1.04 (1.01–1.07) | 1.35 (1.29–1.41) | 1.18 (1.12–1.24) | 1.42 (1.38–1.47) |  |
| Ever | 1 (reference) | 0.98 (0.93–1.04) | 1.32 (1.19–1.46) | 1.18 (1.08–1.30) | 1.44 (1.35–1.53) |  |
| Alcohol consumption |  |  |  |  |  | 0.121 |
| Nondrinker | 1 (reference) | 1.03 (1.00–1.05) | 1.33 (1.27–1.39) | 1.17 (1.11–1.23) | 1.41 (1.37–1.46) |  |
| Drinker | 1 (reference) | 1.03 (0.94–1.13) | 1.49 (1.29–1.73) | 1.31 (1.12–1.53) | 1.59 (1.43–1.76) |  |
| Physical activity |  |  |  |  |  | 0.378 |
| Irregular | 1 (reference) | 1.02 (0.99–1.05) | 1.34 (1.29–1.40) | 1.19 (1.13–1.25) | 1.42 (1.38–1.47) |  |
| Regular | 1 (reference) | 1.10 (0.99–1.22) | 1.32 (1.07–1.63) | 1.06 (0.89–1.27) | 1.47 (1.32–1.63) |  |
| Body mass index (kg/m^2^) | |  |  |  |  | 0.002 |
| <25 | 1 (reference) | 1.01 (0.98–1.04) | 1.32 (1.26–1.38) | 1.12 (1.06–1.18) | 1.34 (1.30–1.39) |  |
| ≥25 | 1 (reference) | 0.98 (0.92–1.04) | 1.31 (1.19–1.45) | 1.20 (1.10–1.32) | 1.50 (1.41–1.60) |  |
| Hypertension |  |  |  |  |  | 0.230 |
| No | 1 (reference) | 1.05(1.01–1.10) | 1.40 (1.30–1.50) | 1.23 (1.12–1.36) | 1.39 (1.30–1.48) |  |
| Yes | 1 (reference) | 1.01 (0.98–1.05) | 1.31 (1.25–1.38) | 1.16 (1.10–1.22) | 1.43 (1.38–1.48) |  |
| Dyslipidemia |  |  |  |  |  | 0.016 |
| No | 1 (reference) | 1.01 (0.98–1.04) | 1.34 (1.28–1.41) | 1.19 (1.12–1.26) | 1.37 (1.32–1.43) |  |
| Yes | 1 (reference) | 1.07 (1.02–1.12) | 1.35 (1.25–1.46) | 1.19 (1.11–1.27) | 1.51 (1.44–1.58) |  |
| Chronic kidney disease |  |  |  |  |  | 0.031 |
| No | 1 (reference) | 1.03 (1.00–1.06) | 1.35 (1.29–1.42) | 1.19 (1.13–1.25) | 1.38 (1.33–1.43) |  |
| Yes | 1 (reference) | 1.02 (0.97–1.08) | 1.32 (1.23–1.43) | 1.16 (1.07–1.26) | 1.50 (1.43–1.57) |  |
| Disability |  |  |  |  |  | 0.589 |
| No | 1 (reference) | 1.03 (1.00–1.06) | 1.34 (1.28–1.41) | 1.17 (1.11–1.24) | 1.41 (1.36–1.46) |  |
| Yes | 1 (reference) | 1.02 (0.97–1.08) | 1.35 (1.24–1.47) | 1.19 (1.09–1.30) | 1.47 (1.40–1.55) |  |
| Number of anti-dementia medication | |  |  |  |  | 0.036 |
| <2 | 1 (reference) | 1.03 (1.00–1.06) | 1.38 (1.32–1.45) | 1.19 (1.13–1.25) | 1.44 (1.39–1.48) |  |
| ≥2 | 1 (reference) | 1.01 (0.95–1.08) | 1.16 (1.05–1.28) | 1.13 (1.01–1.26) | 1.38 (1.29–1.47) |  |
| Abbreviations: HR, hazard ratio; CI, confidence interval; DM, diabetes mellitus.  ^*^HRs (95% CIs) were calculated using a multivariable Cox hazards regression model adjusted for age, sex, place of residence, income, smoking status, alcohol consumption, physical activity, body mass index, hypertension, dyslipidemia, chronic kidney disease, disability, and number of anti-dementia medication. | | | | | | |

Supplemental Table S4. Additional subgroup analysis in individuals with vascular dementia

| Subgroup | Normoglycemia | Prediabetes^*^ | New-onset DM^*^ | DM <5 years^*^ | DM ≥5 years^*^ | *P* for  interaction |
| --- | --- | --- | --- | --- | --- | --- |
| Place of residence |  |  |  |  |  | 0.275 |
| Urban | 1 (reference) | 1.11 (0.98–1.25) | 1.61 (1.34–1.94) | 1.31 (1.10–1.56) | 1.65 (1.47–1.86) |  |
| Rural | 1 (reference) | 1.04 (0.96–1.12) | 1.39 (1.22–1.57) | 1.10 (0.97–1.25) | 1.46 (1.35–1.59) |  |
| Income |  |  |  |  |  | 0.009 |
| Others | 1 (reference) | 1.11 (1.03–1.19) | 1.48 (1.31–1.66) | 1.24 (1.11–1.39) | 1.50 (1.39–1.62) |  |
| Low | 1 (reference) | 0.90 (0.78–1.04) | 1.36 (1.08–1.70) | 0.92 (0.74–1.16) | 1.59 (1.38–1.84) |  |
| Smoking status |  |  |  |  |  | 0.429 |
| Never | 1 (reference) | 1.04 (0.97–1.12) | 1.46 (1.29–1.64) | 1.14 (1.01–1.29) | 1.47 (1.36–1.59) |  |
| Ever | 1 (reference) | 1.12 (0.97–1.29) | 1.43 (1.14–1.79) | 1.24 (1.02–1.51) | 1.69 (1.48–1.94) |  |
| Alcohol consumption |  |  |  |  |  | 0.442 |
| Nondrinker | 1 (reference) | 1.05 (0.98–1.13) | 1.42 (1.28–1.59) | 1.14 (1.02–1.26) | 1.51 (1.41–1.62) |  |
| Drinker | 1 (reference) | 1.14 (0.90–1.45) | 1.77 (1.24–2.52) | 1.56 (1.10–2.20) | 1.67 (1.30–2.15) |  |
| Physical activity |  |  |  |  |  | 0.875 |
| Irregular | 1 (reference) | 1.05 (0.99–1.13) | 1.44 (1.30–1.60) | 1.16 (1.05–1.29) | 1.53 (1.43–1.64) |  |
| Regular | 1 (reference) | 1.09 (0.84–1.41) | 1.62 (1.02–2.58) | 1.18 (0.76–1.85) | 1.38 (1.06–1.79) |  |
| Body mass index (kg/m^2^) | |  |  |  |  | 0.530 |
| <25 | 1 (reference) | 1.00 (0.93–1.07) | 1.36(1.21–1.54) | 1.13 (1.00–1.27) | 1.45(1.34–1.56) |  |
| ≥25 | 1 (reference) | 1.13 (0.97–1.31) | 1.58 (1.26–1.97) | 1.15 (0.93–1.42) | 1.60 (1.39–1.85) |  |
| Hypertension |  |  |  |  |  | 0.014 |
| No | 1 (reference) | 1.07(0.95–1.21) | 1.64 (1.37–1.97) | 1.53 (1.22–1.92) | 1.36 (1.15–1.61) |  |
| Yes | 1 (reference) | 1.05 (0.97–1.13) | 1.37 (1.20–1.55) | 1.10 (0.98–1.23) | 1.54 (1.43–1.66) |  |
| Dyslipidemia |  |  |  |  |  | 0.028 |
| No | 1 (reference) | 1.08 (0.99–1.17) | 1.46 (1.29–1.66) | 1.35 (1.17–1.54) | 1.47 (1.34–1.61) |  |
| Yes | 1 (reference) | 1.02 (0.91–1.14) | 1.41 (1.16–1.72) | 1.00 (0.86–1.17) | 1.55 (1.40–1.72) |  |
| Chronic kidney disease |  |  |  |  |  | 0.230 |
| No | 1 (reference) | 1.07 (0.99–1.15) | 1.44 (1.27–1.63) | 1.20 (1.07–1.35) | 1.45 (1.34–1.58) |  |
| Yes | 1 (reference) | 1.03 (0.90–1.17) | 1.47 (1.21–1.79) | 1.07 (0.88–1.31) | 1.63 (1.45–1.83) |  |
| Disability |  |  |  |  |  | 0.609 |
| No | 1 (reference) | 1.06 (0.97–1.15) | 1.50 (1.31–1.71) | 1.18 (1.03–1.35) | 1.58 (1.45–1.73) |  |
| Yes | 1 (reference) | 1.06 (0.95–1.18) | 1.37 (1.16–1.63) | 1.14 (0.97–1.33) | 1.43 (1.29–1.59) |  |
| Number of anti-dementia medication | |  |  |  |  | 0.030 |
| <2 | 1 (reference) | 1.06 (0.99–1.13) | 1.39 (1.24–1.56) | 1.21(1.09–1.35) | 1.54 (1.43–1.66) |  |
| ≥2 | 1 (reference) | 1.05 (0.88–1.25) | 1.83 (1.41–2.36) | 0.86 (0.62–1.18) | 1.36 (1.12–1.65) |  |
| Abbreviations: HR, hazard ratio; CI, confidence interval; DM, diabetes mellitus.  ^*^HRs (95% CIs) were calculated using a multivariable Cox hazards regression model adjusted for age, sex, place of residence, income, smoking status, alcohol consumption, physical activity, body mass index, hypertension, dyslipidemia, chronic kidney disease, disability, and number of anti-dementia medication. | | | | | | |
